# *CORR* Insights^®^: What Injury Mechanism and Patterns of Ligament Status Are Associated With Isolated Coronoid, Isolated Radial Head, and Combined Fractures?

**DOI:** 10.1007/s11999-017-5384-8

**Published:** 2017-05-31

**Authors:** W. Angus Wallace

**Affiliations:** University of Nottingham, Queen’s Medical Centre, Nottingham, NG7 2UH UK

## Where Are We Now?

In the current study, Rhyou and colleagues retrospectively studied 54 patients with surgically-treated elbow fractures. Patients were grouped by those with isolated coronoid fractures, isolated radial head fractures, and injuries in which both the coronoid and the radial head were fractured. In all patients, the coronoid process and/or radial head fractures were visualized using three-dimensional-CT. The authors then used MRI to identify both the cartilaginous and the ligament injuries. The injured ligaments were confirmed at the time of subsequent open surgery.

The value of MRI in identifying the ligament injuries and the correlation of the ligament injuries with bony injuries fits with the findings of earlier studies [1–3]. In a paper published in 2014, Rhyou and colleagues [5] reported their outcomes in 18 patients treated surgically for this group of injuries. The study found that the varus stress test under fluoroscopy with forearm pronation was useful in determining the necessity of repairing the lateral ulnar collateral ligament.

In the current study, Rhyou and colleagues describe these mechanisms with even greater clarity using detailed MRIs and CTs, and their study is a valuable addition to our understanding of ligament injuries.

## Where Do We Need To Go?

The biggest challenge for the treating surgeon, who may not be a specialist, is recognizing these complex injuries at an early stage so that appropriate imaging is carried out. A fall onto the outstretched arm resulting in an elbow injury with immediate pain and/or swelling calls for a careful elbow assessment and good initial imaging. Having identified the injuries and confirmed the mechanism of injury, we are still faced with determining which of these injuries would benefit from open surgical repair and which can be treated without surgery.

Some recommend early—almost immediate—ROM, after surgery [4]. In order to achieve this, a strong and durable collateral ligament reconstruction is called for, with devices that can be well-fixed and can handle load at the end of the operation. Although both autograft and allograft tendons are currently used extensively for collateral ligament repair, providing a well-fixed tendon is rarely achievable because the fixation is not sufficiently stable to allow immediate unprotected movement.

The concept of using artificial devices as scaffolds, however, is starting to emerge. A recent study of explanted polyester devices by Kocsis and colleagues [2] has shown both in-growth and on-growth of fibrous tissue leading to a device that is encased by natural tissue and becomes a biological structure during the months and years after its insertion. This concept may also be applicable to the collateral ligaments of the elbow.

## How Do We Get There?

Orthopaedic surgeons need to more appropriately assess and investigate the painful and/or swollen elbow following trauma. By including more training on elbow trauma management in resident training programs, future general orthopaedic surgeons will have a higher index of suspicion for these injuries. Although radial head +/− coracoid fractures are a “specialist” injury, the condition must be identified by the general orthopaedist first, and if instability is suspected, consideration should be given to referring to an appropriate specialist early.

Unfortunately, the rarity of unstable elbow injuries, particularly those with coronoid fractures, make it impossible to carry out randomized controlled trials. Still, by encouraging collaboration among elbow specialists who are addressing a number of these challenging injuries around the world, and by fostering even closer links between the American Shoulder and Elbow Surgeons, the British Elbow and Shoulder Society, the European Society for Surgery of the Shoulder and the Elbow, and our colleagues in South East Asia and Australia, it would be possible to create a “register” of patients treated surgically. Treatment results from such a register would be eminently publishable, and would represent a valuable contribution to the elbow surgery community.
